# A case of non-neutropenic invasive pulmonary aspergillosis under immune checkpoint inhibitor therapy for malignant melanoma

**DOI:** 10.1016/j.rmcr.2022.101627

**Published:** 2022-03-11

**Authors:** Yoshinori Uchida, So Shimamura, Shuichiro Ide, Kazuki Masuda, Masafumi Saiki, Yusuke Sogami, Hiroshi Ishihara

**Affiliations:** University of Yamanashi Hospital, Department of Internal Medicine 2, Shimokato 1110, Chuo-shi, Yamanashi, 409-3898, Japan

**Keywords:** Invasive pulmonary aspergillosis, Immune checkpoint inhibitors, Air crescent sign, *Aspergillus*, Non-neutropenic, Chronic obstructive pulmonary disease, IPA, Invasive pulmonary aspergillosis, ICIs, immune checkpoint inhibitors, DM, diabetes mellitus, ICS, inhaled corticosteroids, CT, computed tomography, COPD, chronic obstructive pulmonary disease, X-rays, chest radiographs

## Abstract

The patient was a 70-year-old man with diabetes mellitus, alcoholic liver disease and bronchial asthma treated with corticosteroid and long-acting β-agonist inhalants. He had also been treated with nivolumab for advanced malignant melanoma for two years with a partial response. He presented to our department with intractable cough, which was attributed to uncontrolled bronchial asthma. Two weeks later, he presented with a high fever and worsened cough. He was diagnosed with bacterial pneumonia based on severe inflammation revealed by laboratory tests and right upper lung consolidation on chest radiography. Antibiotics via either oral or parenteral administration were ineffective and no pathogen was detected in sputum or blood cultures. Based on the air-crescent sign observed on chest computed tomography and a diffuse pseudomembranous lesion on the airway epithelium that was observed via bronchoscopy along with positive serum Aspergillus antigen, a clinical diagnosis of invasive pulmonary aspergillosis (IPA) was made and liposomal amphotericin B was initiated. Three days later, the patient developed massive hemoptysis, and he died of respiratory failure. Later, aspergillus-like mycelia were observed in the pathology of bronchial biopsy, supporting the clinical diagnosis of IPA. Although the use of immune checkpoint inhibitors has been reported to be beneficial for patients with some infectious diseases, it does not seem to be the case for patients with other infectious diseases including our patient.

## 1 Introduction

Invasive pulmonary aspergillosis (IPA) is a rapidly progressive and fatal disease that classically occurs in immunocompromised hosts but can also occur in non-immunocompromised hosts [1]. The characteristic imaging findings, including some unique bronchoscopic findings of IPA, can be useful for its diagnosis [1,2]. There have been some reports describing the use of immune checkpoint inhibitors (ICIs) is helpful in the treatment of infectious diseases associated with *Aspergillus* [3,4], but some cases of lung *Aspergillus* infection have been reported to be worsened by the use of ICIs [5,6]. Uchida et al. have suggested that ICIs can reactivate T-cell-mediated underlying diseases involving infections and granulomas, resulting in an immune reconstitution syndrome-like response [6]. We report a fatal case of IPA in a mildly immunocompromised patient who had been treated with nivolumab for advanced malignant melanoma.

## 2 Case presentation

**Fig. 1:**
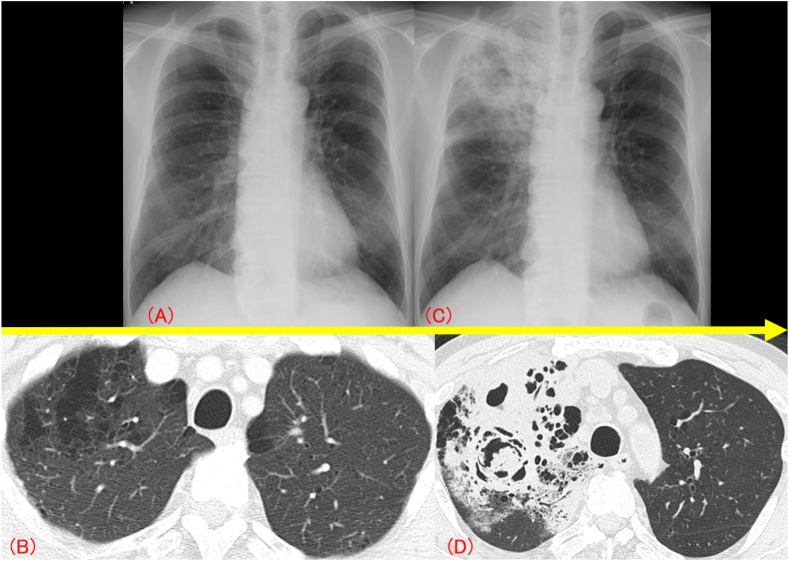
Chest radiographs (X-rays) and computed tomography (CT). On the patient's first presentation, his chest X-ray (A) and chest CT (B) revealed a diffuse emphysematous lesion without any infiltration. At the second visit, his X-ray (C) showed consolidation with a cavity in the right upper lung field, and chest CT (D) showed consolidation with an air-crescent sign.

**Fig. 2:**
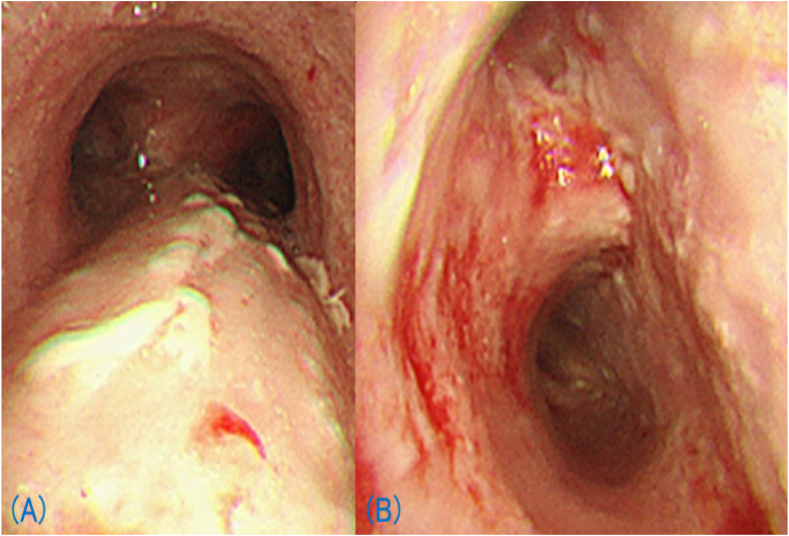
Bronchoscopic findings of airway epithelium. In the trachea (A), a white-coated epithelium suggestive of a pseudomembranous lesion was observed. At the bifurcation of the right upper lobe bronchus and the intermediate truncus (B), white-coated epithelium was found to bleed easily.

**Fig. 3:**
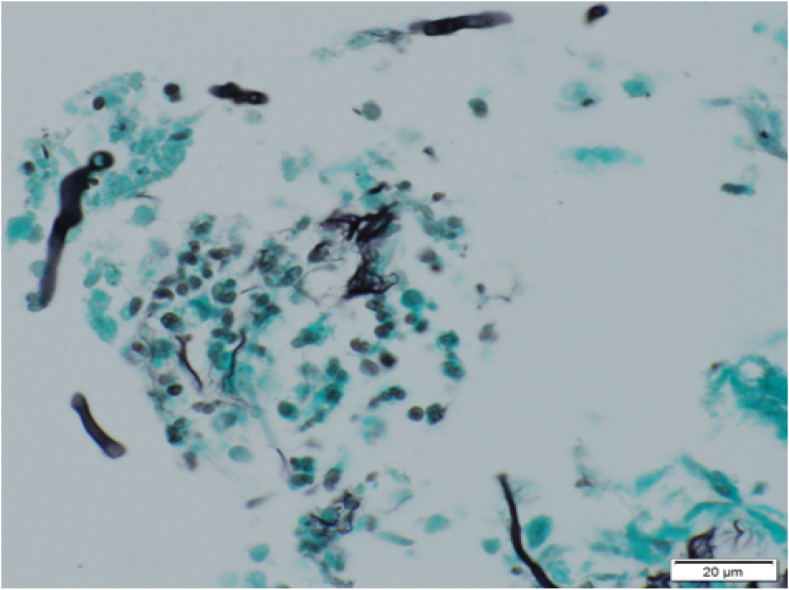
Histopathological finding of bronchial biopsy. In this high-power image with Grocott staining, aspergillus-like mycelia can be observed.

A 70-year-old man with diabetes mellitus (DM), and alcoholic liver disease presented to our department with worsening cough on August 14, 20XX. He had also been treated with nivolumab for malignant melanoma with multiple lung metastases for two years with a partial response prior to his presentation to our department. The cough appeared after two months after nivolumab initiation, and at that time, he was diagnosed with bronchial asthma due to a history of childhood asthma and peripheral blood eosinophilia. Inhaled corticosteroids (ICS)/long-acting beta-agonists had been initiated, but the cough persisted. On presentation, physical examination and laboratory findings did not indicate any infection, and his chest computed tomography (CT) scan showed a diffuse emphysematous lesion without any infiltrations (Fig. 1A and B). A tiotropium oral inhalation was added, but the symptoms were not relieved. On August 28, he again presented with a one-week-long high fever (>40 °C) and worsened cough. Laboratory findings showed severe inflammation with a white blood cell count of 20,560/μL and a C-reactive protein level of 28.34 mg/dL. Chest radiography showed consolidations in the right upper lung (Fig. 1C), and CT scans revealed consolidation with an air-crescent sign (Fig. 1D). A temporal diagnosis of bacterial pneumonia was made, but oral antibiotic treatment was ineffective. The patient was hospitalized four days later, and the antibiotics were changed to tazobactam/piperacillin hydrate, and then to meropenem hydrate + garenoxacin mesylate, though no improvement was observed. No pathogen was detected in sputum or blood culture, but serum Aspergillus antigen was ≥5.0. However, he was negative for β-D glucan and Aspergillus sedimentation antibodies. The QuantiFERON® TB GOLD PLUS result was inconclusive. Bronchoscopy performed on the sixth hospital day showed a diffuse pseudomembranous lesion on the airway epithelium (Fig. 2A and B). A clinical diagnosis of IPA was made, and liposomal amphotericin B was started, but the patient's respiratory condition worsened. Three days after the bronchoscopy, the patient developed massive hemoptysis, and he died of respiratory failure. An autopsy was not performed. Later it was reported that Aspergillus antigen in bronchoalveolar lavage fluid was ≥5.0, and β-D glucan was 52.1 pg/mL. Although the pathogen could not be identified in the culture of the bronchial washing fluid or tissue specimens, aspergillus-like mycelia were observed in the pathology of bronchial biopsy (Fig. 3), supporting the clinical diagnosis of IPA.

## 3 Discussion

Because IPA most often develops in patients in a markedly immunocompromised state, such as neutropenia during chemotherapy for hematologic malignancies, host factors are emphasized in the diagnosis of IPA, as is seen in the European Organization for Research and Treatment of Cancer/Mycosis Study Group criteria [7]. However, it is increasingly being recognized that IPA can also develop in patients in non-neutropenic, mildly immunocompromised states, such as chronic obstructive pulmonary disease (COPD). Therefore, diagnostic criteria for IPA with COPD have been proposed, in which host factors, such as severe immunosuppression, are not required [1]. Although our patient had not been diagnosed with COPD, the IPA diagnosis based on the latter criteria seems to be feasible because of his heavy smoking history and emphysematous lung lesion demonstrated by his chest CT scan. Risk factors for the development of IPA include a high dose of systemic corticosteroid, use of ICS, and preceding viral infection such as influenza. Our patient used inhaled corticosteroids for the treatment of bronchial asthma, which might have predisposed him to *Aspergillus* infection.

In addition to host factors, unique imaging signs of neutropenic IPA, such as the halo sign and air-crescent sign, are helpful for the diagnosis [2]. In non-neutropenic IPA, these signs have been thought to be less frequently observed [1], but recent studies have shown that in patients with IPA and COPD, an air-crescent sign is almost as frequently observed as in neutropenic IPA [8,9], and the diagnosis seems to be more dependent on this sign. Chen et al. reported that two consecutive chest CT scans are useful in the diagnosis of non-neutropenic IPA because cavity formation becomes apparent in the second CT, even though the first CT only demonstrates non-specific findings [10]. They also noted that the time period for cavities to appear was approximately nine days, which is shorter than that of neutropenic IPA [11]. Our patient showed an air-crescent sign on CT imaging obtained one week after symptom onset, and the time period seemed to be long enough for CT imaging to become pathognomonic.

Pseudomembranous epithelial lesions of the airway is also a unique finding of invasive tracheobronchial aspergillosis, which is occasionally accompanied by IPA [1]. Interestingly, Ohta et al. reported a case of invasive tracheobronchial aspergillosis with antedated parenchymal fungal involvement [12]. Accordingly, it is conceivable that the refractory cough in our patient might have been caused by a preceding tracheobronchial *Aspergillus* infection. Alternatively, it might have been an intractable asthma due to *Aspergillus* sensitization or seropositive allergic bronchopulmonary aspergillosis.

With the increased use of ICIs, there have been some reports describing the use of ICIs that are helpful in the treatment of infectious diseases, including *Aspergillus* infection [3]. On the other hand, some cases of lung infections, such as tuberculosis and *Aspergillus* infection, were reportedly worsened by the use of ICIs [5,6,13]. As we have seen in Pneumocystis pneumonia and coronavirus disease 2019 pneumonia, anti-inflammatory therapy is beneficial in the treatment of these diseases. Accordingly, it is conceivable that immune potentiation by ICIs could have negative effects on the outcome of these diseases. The rapid clinical course and poor outcome of our patient did not seem to imply that nivolumab was beneficial for *Aspergillus* infection. Considering that the main pathology of non-neutropenic IPA is inflammatory necrosis rather than angioinvasion and intra-alveolar hemorrhage, which are observed in neutropenic IPA [14], the use of ICIs seemed to negatively affect our patient by potentiating the inflammatory process. Further research and clinical experience are needed to elucidate the potential benefits and dangers of ICIs in the course of infectious diseases.

## 4 Conclusion

We experienced a fatal case of IPA in a patient presenting a mildly immunocompromised non-neutropenic state with inflammatory necrosis, which might have been induced by an increased inflammatory process due to the use of ICIs.

## Funding sources

This research did not receive any grant support.

## Author's contributions

Y.U., S.I., and Y.S. examined the patient; Y.U., S.S., S.I., K.M., M.S., Y.S, and H.I. analyzed the clinical data; Y.U., H.I. wrote the manuscript, which was reviewed and edited by the other coauthors.

## Patient consent for publication

Informed consent was obtained from the patient.

## Declaration of competing interest

The authors have no conflicts of interest to disclose.

## Ethical statement

All medical procedures performed adhered to the tenets of the Declaration of Helsinki.
